# Risk factors for delayed bleeding by onset time after endoscopic submucosal dissection for gastric neoplasm

**DOI:** 10.1038/s41598-019-39381-1

**Published:** 2019-02-25

**Authors:** Hyeong Seok Nam, Cheol Woong Choi, Su Jin Kim, Hyung Wook Kim, Dae Hwan Kang, Su Bum Park, Dae Gon Ryu

**Affiliations:** Department of Internal Medicine, Pusan National University School of Medicine and Research Institute for Convergence of Biomedical Science and Technology, Pusan National University Yangsan Hospital, Yangsan, Korea

## Abstract

Post-endoscopic submucosal dissection bleeding (PEB) is one of the important complications after endoscopic submucosal dissection (ESD), but still difficult to predict. The present study aimed to identify significant risk factors for PEB according to onset time. Between November 2008 and January 2016, a total of 1864 lesions resected via ESD were analyzed. PEB was classified as either early or late according to onset time (within or after 24 hours post-ESD, respectively). During second-look endoscopy, the artificial ulcer bed was subjected to Forrest classification. A high risk of stigma was defined as active spurting bleeding, oozing bleeding, and a non-bleeding visible vessel in the ulcer. The endoscopic factors and medications associated with PEB were analyzed. PEB occurred in 77 lesions (4.1%): early only in 46 (2.4%), late only in 22 (1.1%), and early and late in 9 (0.4%). Among 55 early PEB events, 25 were asymptomatic and diagnosed during second-look endoscopy. Age ≤65 years, resection size ≥30 mm, procedure time ≥20 min, lower third of the stomach, erosion, and clopidogrel use were significantly associated with early PEB. If the number of risk factors were ≤1, the risk of early PEB was 0.6%. For late PEB, the mid to upper third of the stomach, undifferentiated carcinoma, erosion, high risk of stigma during second-look endoscopy, history of early PEB, and clopidogrel use were significant risk factors. If risk factors were absent, the risk of late PEB was 0.1%. For patients at high risk of early PEB, selective second-look endoscopy might be a useful. For patients at high risk of late PEB, careful monitoring of bleeding should be considered.

## Introduction

Endoscopic resection for early gastric cancer (EGC) and adenoma is a well-established treatment modality. The Japanese Gastric Cancer Association recommends endoscopic resection as a first-line treatment for differentiated mucosal cancer lesions ≤20 mm in size without ulceration^1^. Endoscopic submucosal dissection (ESD) for EGC and gastric adenoma has shown a higher en-bloc resection rate and a lower local recurrence rate than conventional endoscopic mucosal resection using an electrosurgical snare^2^. However, the longer procedure time and higher complication rate associated with ESD such as iatrogenic perforation and bleeding are obstacles to the widespread use of ESD^2^. Iatrogenic perforation and long procedure time can be overcome with greater surgical experience and endoscopic instruments. However, post-ESD bleeding (PEB) occurs in 4.5–5.7% of patients^3^.

Intraoperative bleeding during mucosal incision and submucosal dissection is generally not considered a complication until a patient requires a blood transfusion or emergent surgical/radiological intervention. However, delayed PEB detected several hours to days after ESD may result in serious cardiovascular complications. Consensus is lacking on the optimal management of artificial gastric ulcers after ESD such as use of anti-secretory agents (which drugs, optimal doses, and optimal treatment duration) and routine use of second-look endoscopy. Although studies showed that 33–74% of PEB cases occur within 24 hours of ESD^4–6^, several studies of the routine use of second-look endoscopy after ESD showed no advantage for the prevention of PEB^6–9^. However, those studies enrolled relatively a small number of cases and excluded patients taking antiplatelet or antithrombotic medications.

Because unmeasurable factors such as surgical experience, technical factors, and undiagnosed comorbid conditions may be related to PEB, the reported risk factors of PEB are inconsistent^3^. Here we aimed to analyze factors associated with PEB (early within 24 hours and late 24 hours post-ESD) for gastric epithelial neoplasms (EGC and adenoma).

## Materials and Methods

### Patients

**Figure 1:**
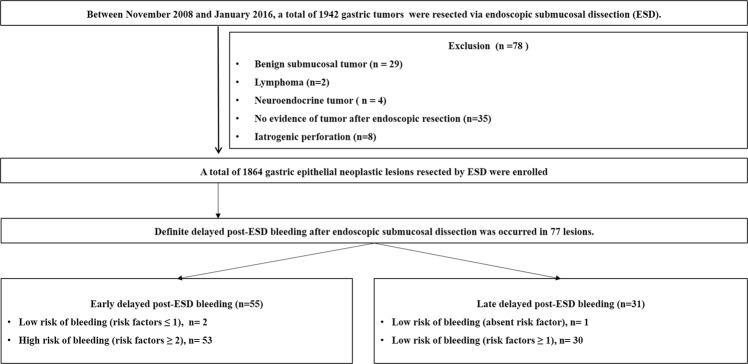
Study flow chart.

Between November 2008 and January 2016, the medical records of patients who underwent ESD at Pusan National University Yangsan Hospital (PNUYH) in the Republic of Korea were reviewed retrospectively. During the study period, a total of 1942 gastric tumors were resected via ESD. Cases of benign submucosal tumor (n = 29), lymphoma (n = 2), neuroendocrine tumor (n = 4), iatrogenic perforation during ESD (n = 8), and no evidence of tumor after ESD (n = 35) were excluded. Finally, a total of 1864 gastric epithelial tumors were enrolled and analyzed (Fig. 1). Written informed consent, including procedure and complications was obtained from all patients before ESD. The present study was approved by the ethics committee of the institutional review board of Pusan National University (L-2018-145).

### Procedure

**Figure 2:**
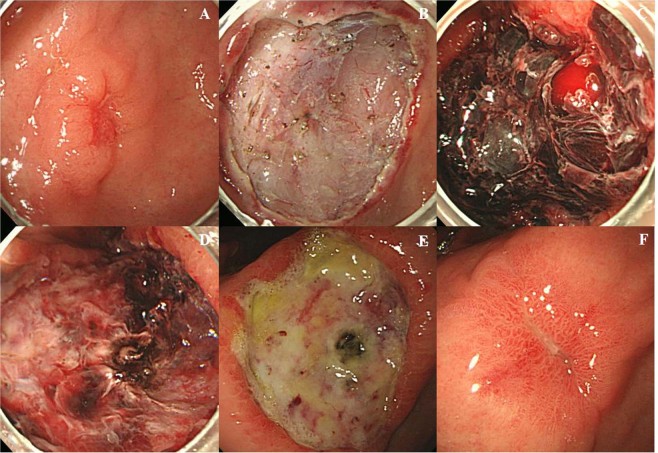
Steps of endoscopic submucosal dissection (ESD). (**A**) diagnostic endoscopy showed a depressed mucosal lesion with central depression and reddish color change at the antrum. (**B**) artificial ulcer just after ESD. (**C**) adherent clot on the artificial ulcer base during second-look endoscopy the day after ESD. (**D**) post-coagulation state during second-look. (**E**) Artificial ulcer bed without bleeding or clots the day after second-look endoscopy. (**F**) artificial ulcer scar after 8 weeks proton pump inhibitor treatment.

A standard single-channel endoscope (GIF-H260, GIF-H260Z, or GIF-HQ290; Olympus Optical, Tokyo, Japan) or a two-channel endoscope (GIF-2TQ260M; Olympus Optical) was used at the surgeons’ discretion. During ESD, after creating a marking of 1–2 mm outside the lesion, a submucosal injection of a solution containing a mixture of normal saline, epinephrine, and indigo carmine was made before the incision or submucosal dissection. A circumferential mucosal incision outside the marking was performed using an electrosurgical generator (ERBEVIO 300D, Endocut I mode, effect 3, duration 2; Erbe Co, Tubingen, Germany). During the submucosal dissection, a coagulation current (Swift coagulation 60 W, ERBE VIO 300D; Erbe Co) was used. After lesion removal, preventive coagulation (Soft coagulation 60 W, ERBE VIO 300D, Erbe Co) was performed of all visible vessels (Fig. 2).

Before the ESD procedure, an intravenous bolus injection of a proton pump inhibitor (pantoprazole 40 mg every 12 hours for 48 hours) was started. We usually recommend discharge 48 hours after ESD when there was no evidence of gastrointestinal bleeding and a regular oral dose of a proton pump inhibitor was started for 8 weeks. When a patient was using an antiplatelet medication (low-dose aspirin or clopidogrel) or antithrombotic medication (warfarin), we consulted the cardiovascular department for guidance on medication management. All patients taking antiplatelet and antithrombotic drugs were asked to stop the medication for 7 days before ESD. For patients using dual antiplatelet or antithrombotic medications, clopidogrel or warfarin was stopped without interruption of the low-dose aspirin. For patients with a high thromboembolic risk, bridging therapy using heparin was performed. All antiplatelet or antithrombotic drugs were restarted 1–2 days post-ESD.

H. pylori diagnosis was performed simultaneously with endoscopic forceps biopsy and rapid urease test at least 2 weeks holding PPI at the 2–6 month follow up endoscopic examination. When both or one of the tests were positive, H. pylori eradication treatment was performed.

### Definition

**Figure 3:**
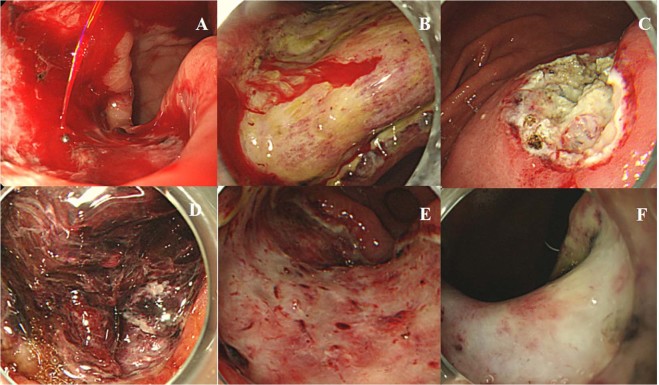
Forrest classification of artificial ulcer after gastric endoscopic submucosal dissection during second-look endoscopy. (**A**) Spurting blood (Ia). (**B**) Oozing blood (Ib). (**C**) Non-bleeding visible vessel (IIa). (**D**) Adherent clot (IIb). (**E**) Flat, pigmented spots (IIc). (**F**) Clean base (III).

Second-look endoscopy was performed in all patients the day after ESD. The high risk of stigma during second-look endoscopy was subjected to Forrest classification (Fig. 3)^10^. During second-look endoscopy, high risk of stigma was defined as spurting blood (Forrest type Ia), oozing blood (Forrest type Ib), and non-bleeding visible vessels (Forrest type IIa). Preventive coagulation was performed for all patients at high risk of stigma on the ulcer bed. We removed adherent clots (Forrest type IIb) and reclassified them according to whether or not there was high risk of stigma. A PEB was defined as clinical evidence of bleeding in the artificial ulcer lesions such as overt hematemesis, melena, spurting or oozing bleeding from the artificial ulcer bed, and the presence of fresh blood or clots in the stomach during endoscopic examination. During second-look endoscopy, oozing blood on the artificial ulcer bed without evidence of blood or clots in the stomach was classified as high risk of stigma rather than PEB. PEB detected during or before the second-look endoscopy within 24 hours was classified as early PEB, whereas that detected after 24 hours was classified as late PEB.

Lesion location was classified as the lower third, middle third, or upper third of the stomach^11^. Each lesion’s maximal diameter was measured via pathologic examination of each resected specimen. Lesion color was compared to the background mucosa. Nodularity was defined as an irregularly raised or nodular mucosal surface. Submucosal fibrosis was recorded after confirming the presence of fibrosis during submucosal dissection. The extent of atrophic gastritis was measured by the endoscopic Kimura–Takemoto classification system: mild (normal to closed type 2), moderate (closed type 3 to open type 1), and severe (open type 2 to open type 3)^12^. The procedure time was calculated from the marking of the lesion to the completion of preventive coagulation of the artificial ulcer bed after lesion removal. Low risk of post-ESD bleeding was defined as the incidence of bleeding being less than 1% according to the number of significant risk factors after multivariate analysis^13^.

The resected specimens were stretched, pinned, and fixed in formalin. Fixed specimens were then sectioned at 2 mm intervals. En-bloc resection was defined as a resection in a single piece of the lesion. Complete resection was defined as the absence of tumor cells at the margins of an en-bloc resected specimen.

### Statistical analyses

Univariate analysis was performed using the chi-square test or Fisher’s exact test for categorical variables or Student’s t-test for continuous variables. The variables with values of p < 0.05 on univariate analysis were included in the multivariate analysis using multiple logistic regression models. Values of p < 0.05 were considered statistically significant. Calculations were performed using the Statistical Package for the Social Sciences (SPSS) version 21.0 for Windows (IBM Corp., Armonk, NY, USA).

### Ethical standard

Written informed consent, including procedure and complications was obtained from all patients before ESD.. The study including the use of patient data was approved by the ethics committee of the Institutional Review Board.

## Results

Figure 1 shows a summary of the final events of PEB. Definite PEB after ESD occurred in 77 lesions (4.1%). Among them, early PEB or late PEB occurred in 55 (2.9%) and 31 patients (1.6%), respectively. In 9 cases (0.4%), early and late PEB occurred in the same lesion. All cases of PEB were successfully treated by endoscopic hemostasis.

The patients’ clinicopathologic characteristics are summarized in Table 1. The mean patient age was 68.3 ± 9.3 years. The mean lesion and resection sizes were 12.6 ± 8.1 mm and 30.2 ± 9.3 mm, respectively. The mean procedure time was 24.9 ± 17.4 min. The most common lesion location was the lower third of the stomach (65.2%). The en-bloc and complete resection rates were 98.4% and 95.4%, respectively. Among the resected lesions, 64.5% were adenoma and 35.5% were adenocarcinoma. Low-dose aspirin, clopidogrel, and warfarin medications were found in 11.4%, 3.8%, and 0.4% of lesions, respectively.

**Table 1:** Baseline patient characteristics.

	Total (n = 1864)
Age, years, mean (SD)	68.3 (9.3)
Male sex, n (%)	1385 (74.3)
Lesion size, mm, mean (SD)	12.6 (8.1)
Resection size, mm, mean (SD)	30.2 (9.3)
Procedure time, min, mean (SD)	24.9 (17.4)
En-bloc resection, n (%)	1834 (98.4)
Histologic complete resection, n (%)	1779 (95.4)
Pathologic diagnosis, n (%)	
Adenoma	1203 (64.5)
Differentiated carcinoma	626 (33.6)
Undifferentiated carcinoma	35 (1.9)
Post-operative bleeding, n (%)	77 (4.1%)
Early post-ESD bleeding	55 (2.9)
Late post-ESD bleeding	31 (1.6)
Early and late post-operative bleeding	9 (0.5)
Aspirin use, n (%)	212 (11.4)
Clopidogrel use, n (%)	71 (3.8)
Warfarin use, n (%)	7 (0.4)
*Helicobacter pylori* infection, n (%)	947 (50.8)

SD, standard deviation.

**Figure 4:**
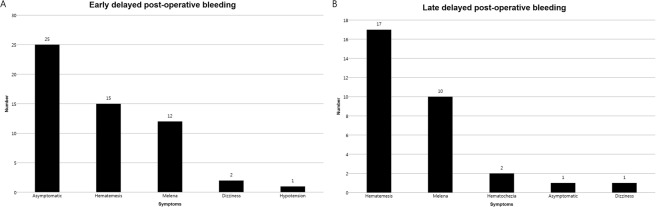
Symptoms of delayed bleeding after gastric endoscopic submucosal dissection. (**A**) Early post-operative bleeding. (**B**) Late post-operative bleeding.

Risk factor analyses associated with early PEB are shown in Tables 2 and 3. On univariate analysis, age ≤65 years, lesion size ≥20 mm, resection size ≥30 mm, procedure time ≥20 min, location in lower third of the stomach, erosion, and clopidogrel use were significant. On multivariate analysis, age ≤65 years (odds ratio [OR], 3.375; 95% confidence interval [CI], 1.919–5.935; p < 0.001), resection size ≥30 mm (OR, 2.027; 95% CI, 1.021–4.026; p = 0.044), procedure time ≥20 min (OR, 2.453; 95% CI, 1.185–5.078; p = 0.016), lower third of the stomach (OR, 2.845; 95% CI, 1.381–5.860; p = 0.005), erosion (OR, 1.870; 95% CI, 1.056–3.313; p = 0.032), and clopidogrel use (OR, 4.041; 95% CI, 1.587–10.286; *p* = 0.003) were significant. The symptoms at the time of PEB diagnosis are shown in Fig. 4A. Common symptoms for early PEB were asymptomatic (n = 25), followed by hematemesis (n = 15) and melena (n = 12). Asymptomatic patients were diagnosed during routine second-look endoscopy.

**Table 2:** Factors associated with early post-operative bleeding; univariate analysis.

	No evidence of early post-operative bleeding (n = 1809)	Early post-operative bleeding (n = 55)	Total (n = 1864)	*P* value
Age ≥65 years, n (%)	1210 (66.9)	23 (41.8)	1233 (66.1)	<0.001
Male sex, n (%)	1342 (74.2)	43 (78.2)	1385 (74.3)	0.504
Lesion size ≥20 mm, n (%)	289 (16.0)	15 (27.3)	304 (16.3)	0.025
Resection size ≥30 mm, n (%)	910 (50.3)	41 (74.5)	951 (51.0)	<0.001
Procedure time ≥20 min, n (%)	1033 (57.1)	44 (80.0)	1077 (57.8)	0.001
Lesion location, n (%)				0.014
Lower third	1171 (64.7)	45 (81.8)	1216 (65.2)	
Middle third	489 (27.0)	10 (18.2)	499 (26.8)	
Upper third	149 (8.2)	0 (0)	149 (8.0)	
Lesions at the lower third of stomach, n (%)	1171 (64.7)	45 (81.8)	1216 (65.2)	0.009
En-bloc resection, n (%)	1790 (98.4)	54 (98.2)	1834 (98.4)	0.901
Final pathologic diagnosis, n (%)				0.439
Adenoma	1171 (64.7)	32 (58.2)	1203 (64.5)	
Differentiated carcinoma	605 (33.4)	21 (38.4)	626 (33.6)	
Undifferentiated carcinoma	33 (1.8)	2 (3.6)	35 (1.9)	
Endoscopic atrophic gastritis, n (%)				0.127
Mild extent	426 (23.5)	18 (32.7)	444 (23.8)	
Moderate extent	857 (47.4)	27 (49.1)	884 (47.4)	
Severe extent	526 (29.1)	10 (18.2)	536 (28.8)	
Morphology of lesions, n (%)				0.146
Elevated	954 (52.7)	22 (40.0)	976 (52.4)	
Flat	245 (13.5)	8 (14.5)	253 (13.6)	
Depressed	610 (33.7)	25 (45.5)	635 (34.1)	
Ulceration, n (%)	84 (4.6)	3 (5.5)	87 (4.7)	0.779
Submucosal fibrosis, n (%)	288 (15.9)	13 (23.6)	301 (16.1)	0.126
Erythema, n (%)	1289 (71.3)	43 (78.2)	1332 (71.5)	0.262
Nodularity, n (%)	453 (25.0)	17 (30.9)	470 (25.2)	0.324
Depression, n (%)	513 (28.4)	18 (32.7)	531 (28.5)	0.479
Erosion, n (%)	467 (25.8)	22 (40.0)	489 (26.2)	0.018
Submucosal invasive lesion, n (%)	93 (5.1)	3 (5.5)	96 (5.2)	0.917
Lymphatic invasion, n (%)	19 (1.1)	0 (0)	19 (1.0)	0.445
Aspirin use, n (%)	207 (11.4)	5 (9.1)	212 (11.4)	0.588
Clopidogrel use, n (%)	65 (3.6)	6 (10.9)	71 (3.8)	0.005
Warfarin use, n (%)	6 (0.3)	1 (1.8)	7 (0.4)	0.076
*Helicobacter pylori* infection, n (%)	915 (96.6)	32 (58.2)	947 (50.8)	0.267

SD, standard deviation.

**Table 3:** Risk factor analysis of early post-operative bleeding after gastric endoscopic submucosal dissection; univariate and multivariate analyses.

	Univariate analysis					
	OR	95% CI	*P* value	OR	95% CI	*P* value
Age ≤ 65 years	2.810	1.630–4.845	<0.001	3.375	1.919–5.935	<0.001
lesion size ≥20 mm	1.924	1.077–3.439	0.025	1.409	0.717–2.771	0.320
Resection size ≥30 mm	2.812	1.543–5.122	<0.001	2.027	1.021–4.026	0.044
Procedure time ≥20 min	2.923	1.519–5.623	0.001	2.453	1.185–5.078	0.016
Lower third of stomach	2.398	1.217–4.727	0.009	2.845	1.381–5.860	0.005
Erosion	1.916	1.106–3.319	0.018	1.870	1.056–3.313	0.032
Clopidogrel use	3.285	1.358–7.946	0.005	4.041	1.587–10.286	0.003

OR, odds ratio; CI, confidence interval.

Tables 4 and 5 show the risk factor analysis associated with late PEB. A total of 31 events (1.6%) occurred. On univariate analysis, age ≤65 years, procedure time ≥20 min, mid to upper third of the stomach, undifferentiated carcinoma, erosion, submucosal invasion, high risk of stigma during second-look endoscopy, history of early delayed bleeding, and the use of aspirin, clopidogrel, and warfarin were significant. After multivariate analysis, mid to upper third of the stomach (OR, 4.664; 95% CI, 1.990–10.929; p < 0.001), undifferentiated carcinoma (OR, 4.878; 95% CI, 1.112–21.394; p = 0.036), erosion (OR, 2.819; 95% CI, 1.259–6.310; p = 0.012), high risk of stigma during second-look endoscopy (OR, 4.215; 95% CI, 1.272–13.971, p = 0.019), history of early PEB (OR, 3.899; 95% CI, 1.009–15.064; p = 0.048), and clopidogrel use (OR, 4.257; 95% CI, 1.364–13.288; *p* = 0.013) were significant. The symptoms at the time of late PEB diagnosis are shown in Fig. 4B. Common symptoms were hematemesis (n = 17) and melena (n = 10).

**Table 4:** Factors associated with late post-operative bleeding; univariate analysis.

	No evidence of late post-operative bleeding (n = 1833)	Late post-operative bleeding (n = 31)	Total (n = 1864)	*P* value
Age ≥65 years, n (%)	1218 (66.4)	15 (48.4)	1233 (66.1)	0.035
Male, n (%)	1358 (74.1)	27 (87.1)	1385 (74.3)	0.100
Lesion size ≥20 mm, n (%)	296 (16.1)	8 (25.8)	304 (16.3)	0.149
Resection size ≥30 mm, n (%)	931 (50.8)	20 (64.5)	951 (51.0)	0.130
Procedure time ≥20 min, n (%)	1050 (57.5)	27 (87.1)	1077 (57.8)	0.001
Lesions at lower third of stomach, n (%)	12.4 (65.7)	12 (38.7)	1216 (65.2)	0.002
En-bloc resection, n (%)	1803 (98.4)	31 (100)	1834 (98.4)	0.473
Undifferentiated carcinoma, n (%)	32 (1.7)	3 (9.7)	35 (1.9)	0.001
Endoscopic atrophic gastritis, n (%)				0.583
Mild extent	439 (23.9)	5 (16.1)	444 (23.8)	
Moderate extent	867 (47.3)	17 (54.8)	884 (47.4)	
Severe extent	527 (28.8)	9 (29.0)	536 (28.8)	
Morphologic of lesions, n (%)				0.809
Elevated	959 (52.3)	17 (54.8)	976 (52.4)	
Flat	248 (13.5)	5 (16.1)	253 (13.6)	
Depressed	626 (34.2)	9 (29.0)	635 (34.1)	
Ulceration, n (%)	85 (4.6)	2 (6.5)	87 (4.7)	0.635
Submucosal fibrosis, n (%)	292 (15.9)	9 (29.0)	301 (16.1)	0.049
Erythema, n (%)	1310 (71.5)	22 (71.0)	1332 (71.5)	0.951
Nodularity, n (%)	462 (25.2)	8 (25.8)	470 (25.2)	0.939
Depression, n (%)	524 (28.6)	7 (22.6)	531 (28.5)	0.462
Erosion, n (%)	476 (26.0)	13 (41.9)	489 (26.2)	0.045
Submucosal invasive lesion, n (%)	92 (5.0)	4 (12.9)	96 (5.2)	0.049
Lymphatic invasion, n (%)	19 (1.0)	0 (0)	19 (1.0)	0.569
High risk of stigma during second-look endoscopy, n (%)	163 (8.9)	13 (41.9)	176 (9.4)	<0.001
Early post-operative bleeding, n (%)	46 (2.5)	9 (29.0)	55 (3.0)	<0.001
Aspirin use, n (%)	205 (11.2)	7 (22.6)	212 (11.4)	0.047
Clopidogrel use, n (%)	65 (3.5)	6 (19.4)	71 (3.8)	<0.001
Warfarin use, n (%)	6 (0.3)	1 (3.2)	7 (0.4)	0.009
*Helicobacter pylori* infection, n (%)	928 (50.6)	19 (61.3)	947 (50.8)	0.239

SD, standard deviation.

**Table 5:** Risk factor analysis of late post-operative bleeding after gastric endoscopic submucosal dissection; univariate and multivariate analyses.

	Univariate analysis			Multivariate analysis		
	OR	95% CI	*P* value	OR	95% CI	*P* value
Age ≤65 years	2.113	1.038–4.301	0.035	1.944	0.884–4.276	0.098
Procedure time ≥20 min	4.932	1.733–14.039	0.001	2.839	0.942–8.552	0.064
Mid to upper third of stomach	3.031	1.462–6.283	0.002	4.664	1.990–10.929	<0.001
Undifferentiated carcinoma	6.030	1.743–20.857	0.001	4.878	1.112–21.394	0.036
Erosion	2.059	1.001–4.234	0.045	2.819	1.259–6.310	0.012
Submucosal invasion	2.804	0.961–8.180	0.049	1.988	0.579–6.816	0.274
High risk of stigma during second-look endoscopy	7.399	3.561–15.374	<0.001	4.215	1.272–13.971	0.019
History of early post-operative bleeding	15.892	6.937–36.407	<0.001	3.899	1.009–15.064	0.048
Aspirin use	2.316	0.986–5.443	0.047	1.990	0.683–5.287	0.218
Clopidogrel use	6.528	2.589–16.459	<0.001	4.257	1.364–13.288	0.013
Warfarin use	10.150	1.185–86.921	0.009	13.242	0.942–186.085	0.055

OR, odds ratio; CI, confidence interval.

**Figure 5:**
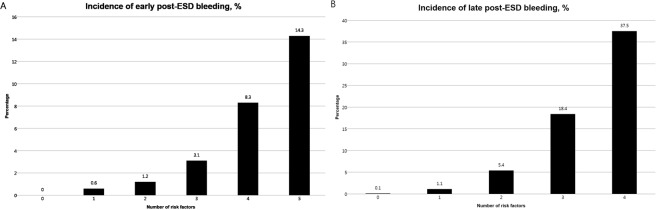
Incidence of post-ESD bleeding according to the number of risk factors. A. early post-ESD bleeding, the risk factors of early post-ESD bleeding include age ≤65 years, resection size ≥30 mm, procedure time ≥20 min, lower third of stomach, erosion, and clopidogrel use. B. late-post ESD bleeding, the risk factors of late post-ESD bleeding include the mid to upper third of the stomach, undifferentiated carcinoma, erosion, high risk of stigma during second-look endoscopy, history of early post-ESD bleeding, and clopidogrel use. ESD; endoscopic submucosal dissection.

According the number of risk factors, the incidence rate of PEB is shown in Fig. 5. The incidence of early PEB was less than 1% if the number risk factor was ≤1. The incidence of late PEB was than 1% if risk factors were absent.

## Discussion

The present study results show a PEB rate of 4.1%. The reported incidence of ESD-related bleeding varies. The reported intra-procedural and PEB rates were 11.9–45.1% and 5.1%, respectively^3,14,15^. Bleeding is inevitable during mucosal incision and submucosal dissection using an electrosurgical knife. Intra-procedural bleeding, manageable by an endoscopic maneuver, should not be considered a complication. A delayed diagnosis of PEB in artificial ulcers after ESD may be accompanied by cardiovascular compromise due to late symptom onset. Among cases of PEB, 33–74% reportedly occur within 24 hours after ESD^4–6^. In the present study, early PEB occurred in 55 patients. In addition, 25 patients were asymptomatic before active bleeding was noted on routine second-look endoscopy. In cases of acute gastrointestinal bleeding, hemoglobin levels do not fall immediately because of a proportionate reduction in plasma and red blood cell volumes. Therefore, hemoglobin levels may be normal or only minimally decreased at the initial presentation of severe artificial ulcer bleeding. Diagnosing or detecting PEB before the onset of cardiovascular compromise is important, especially for older patients and those at risk of cardiovascular morbidity. Recent prospective studies on the efficacy of the routine use of second-look endoscopy showed no advantage for the prevention of PEB^6–9^. However, those studies had several limitations such as differing definitions of PEB, the exclusion of patients who took antiplatelet/antithrombotic medications, and small sample sizes. Therefore, patients at high risk of PEB might have been excluded from those studies. In the present study, we included patients who took antiplatelet/antithrombotic medications (low-dose aspirin, clopidogrel, and warfarin) and analyzed the risk factors associated with early and late PEB.

In the present study, the analysis of risk factors associated with PEB was performed according to early or late onset. Early PEB was associated with age ≤65 years, resection size ≥30 mm, procedure time ≥20 min, location in the lower third of the stomach, erosion, and clopidogrel use. The reason why younger patients are at higher risk of PEB is unclear. Mucosal and submucosal vascular prominence may differ among age groups. For older patients, the vascular plexus may decrease proportionate to age and atrophic mucosal changes^14^. In South Korea, both endoscopic and histological atrophy increased proportionate to age^16^. Longer procedure time and larger resection size may be associated with a more difficult ESD procedure. A difficult ESD procedure may be associated with lesion location, lesion size, anatomical factors, surgical experience, or poor patient cooperation. Damage to vessels beneath the submucosa or proper muscle may occur more frequently during a difficult ESD procedure. Surface erosion of the gastric epithelial tumors is associated with endoscopic findings of higher-grade dysplasia or EGC^17^. Surface erosion may be associated with active inflammation or previous repeated endoscopic forceps biopsy. An active inflammatory lesion may be associated with the existence of more vessels in the submucosal layer^5^.

The PEB tendency differed according to lesion location. In the lower third of the stomach, early PEB occurred more frequently. However, late PEB was more frequently associated with the mid to upper third of the stomach. At the earlier time after ESD, antral contractility and bile or digestive enzyme reflux from the duodenum might be associated with bleeding events. A previous study reported that the preventive coagulation of visible vessels at the artificial ulcer bed just after tumor excision was important in preventing PEB^18^. Intraoperative bleeding occurs less frequently in the antrum than in the mid to upper part of the stomach. Therefore, preventive coagulation might be insufficient during and just after ESD. The submucosal arteries in the mid to upper part of the stomach are more stubby and thicker than those in the antrum^18^. During the ESD procedure, intraoperative bleeding occurs more frequently than in the antrum because of submucosal artery damage. The gastric wall and submucosa thickness differ between anatomical locations. The body wall and submucosa are thinner than the antrum^19^. Endoscopic handling is more difficult in the mid to upper third of the stomach than in the antrum. Preventive coagulation at the ulcer bed for exposed or bleeding vessels might be insufficient during the ESD procedure. Undifferentiated carcinoma was a risk factor for late PEB. Undifferentiated carcinoma occurs more frequently in the mid to upper part of the stomach than in the antrum^20^.

In the present study, clopidogrel use was an important risk factor for early and late PEB. We asked our patients to stop taking clopidogrel 7 days before ESD and to restart it 1–2 days after ESD. Clopidogrel is a potent antiplatelet inhibitor that decreases ulcer-induced gastric epithelial cell proliferation and inhibits angiogenesis in gastric ulcer healing^21,22^. Thus, clopidogrel might inhibit the healing process of artificial ulcers and increase the patient’s risk of PEB.

In the present study, routine second-look endoscopy was performed in all patients the day after ESD. We subjected the artificial ulcer bed to Forrest classification, which is used for risk classification of peptic ulcer re-bleeding. For patients at high risk of stigma ulcer, additive preventive endoscopic hemostasis was performed. The high risk of stigma and early PEB lesions were risk factors for late PEB despite additive hemostasis. Previous studies have reported that patients who underwent second-look endoscopy showed higher bleeding rates than patients in non-second look endoscopic groups^6,7^. Another study reported that routine prophylactic hemostasis during second-look endoscopy may be ineffective for preventing PEB^23^. The reason for this is unclear. However, incomplete hemostasis for the exposed vessel or active bleeding spots might be one possible reason. In addition, additive coagulation for patients at low risk of bleeding ulcer (such as oozing bleeding or pigmentation) may aggravate tissue and vessel damage beneath the submucosa.

In the present study, the incidence rate of PEB according to the number of risk factors was calculated. The results of present study highlighted that patients with early PEB with ≥2 risk factors had a PEB rate of more than 1.2% within 24 hours of ESD. The incidence rate increased in line with increasing risk factor numbers. Therefore, these patients may be candidates for second-look endoscopy. Patients with late PEB with ≥1 risk factor, the risk of late PEB was more than 1.1% and the increasing incidence of bleeding was associated with increasing risk factor numbers. These patients should be recommended for careful monitoring after discharge.

The present study has several limitations. First, selection bias may be present because of a retrospective analysis of medical chart review. Therefore, we cannot generalize the present study results. Second, in the present study, we could only evaluate low-dose aspirin, clopidogrel, and warfarin from the retrospective medical chart review. Direct-acting anticoagulants (DOAC) could not be confirmed in this study. Several other antiplatelet medications, antithrombotic medications, and other unchecked medications might be associated with bleeding events. Third, although 7 dialysis patients and 8 atrial fibrillation patients were included, the patients’ comorbid diseases associated with bleeding tendency could not be evaluated from the retrospective chart review. Fourth, the effect of second-look endoscopy could not be evaluated because all patients underwent second-look endoscopy.

In summary, age ≤65, resection size ≥30 mm, procedure time ≥20 min, location in the lower third of the stomach, erosion, and clopidogrel use were associated with early PEB. Routine second-look endoscopy for all patients might not be beneficial. However, the incidence of early PEB is increased according the number of risk factors. Therefore, patients with ≥2 risk factors may be considered for second-look endoscopy. Location in the mid to upper third of the stomach, undifferentiated carcinoma, erosion, clopidogrel use, high risk of stigma, and history of early PEB were associated with late PEB. After patients with these risk factors are discharged, careful monitoring should be considered.
